# Editorial: Monogenic diabetes: from genetics and cell biology to clinical practice

**DOI:** 10.3389/fendo.2022.1022611

**Published:** 2022-08-31

**Authors:** Li Ding, Fabrizio Barbetti, Ming Liu

**Affiliations:** ^1^ Department of Endocrinology and Metabolism of Tianjin Medical University General Hospital, Tianjin, China; ^2^ Department of Experimental Medicine, University of Rome Tor Vergata, Rome, Italy

**Keywords:** monogenic diabetes, maturity-onset diabetes of the young, personalized medicine, clinical characterisation, pathogenesis

Monogenic diabetes mellitus (MDM) is a special type of diabetes caused by a single gene mutation. MDM accounts for approximately 1-5% of all causes of diabetes. MDM can be classified as neonatal diabetes mellitus (NDM), maturity-onset diabetes of the young (MODY), mitochondrial diabetes, and some rare genetic syndromes associated with diabetes. To date, mutations in more than 60 genes have been reported to cause MDM. Most of the genes are critical for pancreatic beta cell function, development, and survival. Pathogenetic mutations in the genes involved in insulin action can also cause MDM. Over the past decades, significant advances have been made in MDM awareness, diagnosis, and management. However, patients with MDM are easily misdiagnosed as type 1 diabetes or type 2 diabetes, and may not receive appropriate management. There are significant unmet medical needs for improving recognition, appropriate clinical/genetic testing/diagnosis and management, as well as better understanding of the underlying mechanisms of MDM. In this special issue, we have come together an array of reports encompassing disease spectrum and clinical characteristics of monogenic diabetes, as well as novel techniques for functional analysis of variants.

A series of studies in this Research Topic expanded our knowledge regarding genetic etiology and pathogenesis of monogenic diabetes. Yang et al. identified a novel, heterozygous INS nonsense mutation, preproinsulin R46X in two unrelated patients with early onset diabetes. Mechanistically, despite with an intact signal peptide, R46X failed to be efficiently translocated into the ER, highlighting that proinsulin domain downstream of signal peptide plays an important unrecognized role in preproinsulin translocation. Given the fact that some family members carrying R46X do not develop diabetes, further studies are warranted to determine additional genetic and/or environmental factors contributing to the development and progression of diabetes in the patients carrying R46X. In an intriguing case report, Zhou et al. described a Chinese family of Woodhouse-Sakati syndrome presented with diabetes mellitus, hypogonadotropic hypogonadism, central hypothyroidism, alopecia, and intellectual disability, caused by a novel biallelic deletion mutation of the DCAF17 gene. Jiang et al. identified a novel missense mutation GCK p.A259T, which cosegregated with diabetes in a Chinese MODY2 pedigree. The authors conducted extensive kinetic and thermal stability analysis, and showed that the mutation impaired the affinity, catalytic capability, and cooperativity for the glucose substrate. Koneshamoorthy et al. described a novel GCK activating mutation in an adult with hypoglycemia. The variant segregated with hypoglycemia in the pedigree, and morphological changes were observed in the islet of the patient, along with accentuated glucose stimulated insulin secretion, cytosolic calcium response to glucose, and changes in single cell transcriptomics in the pancreatic islets.

Functional analysis of variants identified is vital in confirmation of disease causality and pursuit of treatment options, but can be difficult. In a two-part series, Dhayalan et al. proposed a single-chain peptide model, “DesDi”, that comprised 49 residues and a single disulfide bridge, optimized for efficiency of disulfide pairing. The platform enabled comparative biophysical studies, regarding ɑ-helix contents, thermal unfolding profiles, thermodynamic stabilities, and NMR resonance properties, of mutations that cause Mutant INS-gene Induced Diabetes of the Young (MIDY). Location and degree of structural perturbation that the MIDY variants caused, assessed by the peptide model, correlated with degree of ER stress and age of diabetes onset. Taken together, the model allows molecular dissection of phenotype-genotype relationships of MIDY variants. Guo et al. developed a scalable dual fluorescence assay in cells, that enables functional characterization of HNF‐4α missense variants, with respect to transcription activity and expression abundance, identified in exome sequencing. In combination with cloning and Sanger sequencing, the method allows quantitative and high-throughput interpretation of HNF‐4α variants.

Monogenic diabetes is under-diagnosed. Awareness of clinical presentations of monogenic diabetes is a prerequisite for recognition of the condition, and subsequent diagnosis and personalized management. In a comprehensive review, Li et al. discussed the pathogenesis and treatment of maturity-onset diabetes of the young type 3, and the association of HNF1A single nucleotide polymorphisms with type 2 diabetes and gestational diabetes. Zhao et al. reviewed the literature extensively, and investigated the clinical presentations of patients with HNF1-alpha MODY. The authors found that clinical manifestations of HNF1-alpha MODY differed by geographical regions and HNF1-alpha mutations. Ge et al. reviewed the clinical presentations and gene mutations of MODY5, in the literature, and concluded that testing for MODY5 should be prioritized in patients with early-onset diabetes, low or normal BMI, renal cysts, hypomagnesemia, and pancreatic dysplasia. Yang et al. reviewed literature, and investigated the genetic spectrum and the clinical features of maternally inherited diabetes and deafness caused by deleterious mitochondrial mutations. The authors found that the heteroplasmy levels of the m.3243A>G mutation in the peripheral blood was negatively correlated with age at the onset of diabetes, and argued that young onset of diabetes with low or normal BMI, maternal inheritance and presence of impairments of multiple systems should prompt a genetic testing for maternally inherited diabetes and deafness. Zhao et al. found that plasma lactate immediately and 30 minutes after exercise could contribute to the differential diagnosis of mitochondrial diabetes and type 1 diabetes. In longitudinal follow-up, fasting C-peptide in patients with mitochondrial diabetes declined rapidly within the first 5 years after diagnosis, and stabilized at low levels 10 years after diagnosis. Abreu et al. investigated the proportion and genetic spectrum of monogenic diabetes in a cohort of patients in Brazil, providing evidence for screening for monogenic diabetes in such a population with mixed ethnic backgrounds.

The field of monogenic diabetes is rapidly evolving. We hope this Research Topic will motivate doctors/researchers in promoting wider recognition, timely diagnosis, and proper management of monogenic diabetes.

## Author contributions

All authors listed have made a substantial, direct, and intellectual contribution to the work and approved it for publication.

## Funding

This work was supported by the National Natural Science Foundation of China 446 (81830025), and also by Tianjin Key Medical Discipline 451 (Specialty) Construction Project (TJYXZDXK-030A).

## Conflict of interest

The authors declare that the research was conducted in the absence of any commercial or financial relationships that could be construed as a potential conflict of interest.

## Publisher’s note

All claims expressed in this article are solely those of the authors and do not necessarily represent those of their affiliated organizations, or those of the publisher, the editors and the reviewers. Any product that may be evaluated in this article, or claim that may be made by its manufacturer, is not guaranteed or endorsed by the publisher.

